# The main protease 3CLpro of the SARS-CoV-2 virus: how to turn an enemy into a helper

**DOI:** 10.3389/fbioe.2023.1187761

**Published:** 2023-06-29

**Authors:** Svetlana V. Belenkaya, Iuliia A. Merkuleva, Olga I. Yarovaya, Varvara Yu. Chirkova, Elena A. Sharlaeva, Daniil V. Shanshin, Ekaterina A. Volosnikova, Sergey Z. Vatsadze, Mikhail V. Khvostov, Nariman F. Salakhutdinov, Dmitriy N. Shcherbakov

**Affiliations:** ^1^ Laboratory of Bionanotechnology, Microbiology and Virology, Novosibirsk State University, Novosibirsk, Russia; ^2^ State Research Center of Virology and Biotechnology VECTOR, Koltsovo, Russia; ^3^ Department of Medicinal Chemistry, N.N Vorozhtsov Novosibirsk Institute of Organic Chemistry SB RAS, Novosibirsk, Russia; ^4^ Department of Physical-Chemistry Biology and Biotechnology, Altay State University, Barnaul, Russia; ^5^ N.D Zelinsky Institute of Organic Chemistry, Russian Academy of Sciences, Moscow, Russia

**Keywords:** *E. coli* bacteria, 3CL protease, TEV protease, RBD, SARS-CoV-2

## Abstract

Despite the long history of use and the knowledge of the genetics and biochemistry of *E. coli*, problems are still possible in obtaining a soluble form of recombinant proteins in this system. Although, soluble protein can be obtained both in the cytoplasm and in the periplasm of the bacterial cell. The latter is a priority strategy for obtaining soluble proteins. The fusion protein technology followed by detachment of the fusion protein with proteases is used to transfer the target protein into the periplasmic space of *E. coli*. We have continued for the first time to use the main viral protease 3CL of the SARS-CoV-2 virus for this purpose. We obtained a recombinant 3CL protease and studied its complex catalytic properties. The authenticity of the resulting recombinant enzyme, were confirmed by specific activity analysis and activity suppression by the known low-molecular-weight inhibitors. The catalytic efficiency of 3CL (0.17 ± 0.02 µM-1-s-1) was shown to be one order of magnitude higher than that of the widely used tobacco etch virus protease (0.013 ± 0.003 µM-1-s-1). The application of the 3CL gene in genetically engineered constructs provided efficient specific proteolysis of fusion proteins, which we demonstrated using the receptor-binding domain of SARS-CoV-2 spike protein and GST fusion protein. The solubility and immunochemical properties of RBD were preserved. It is very important that in work we have shown that 3CL protease works effectively directly in *E. coli* cells when co-expressed with the target fusion protein, as well as when expressed as part of a chimeric protein containing the target protein, fusion partner, and 3CL itself. The results obtained in the work allow expanding the repertoire of specific proteases for researchers and biotechnologists.

## 1 Introduction

*Escherichia coli* (*E. coli*) bacterium is one of the most popular systems for products recombinant proteins in both laboratory research and industrial biotechnology (Wagner et al., 2006; Huang et al., 2012; Rosano and Ceccarelli, 2014; Baeshen et al., 2015). A feature of the *E. coli* expression system is that the synthesized recombinant protein can be accumulated in a soluble or insoluble form. Protein in the insoluble form deposited in the form of so-called inclusion bodies, the large intracellular structures (Rosano et al., 2019). The protein partially loses its functions, and special procedures related to conversion into a soluble state and restoration of the secondary and tertiary protein structure are needed to restore them (Clark, 2001; Li et al., 2004; Nara et al., 2009). The protein in a soluble state can be accumulated in the cytoplasm or periplasm of *E. coli*; less frequently, it is secreted into the culture medium. The most popular strategy for obtaining a soluble recombinant protein with preserved properties is transport into the periplasmic space (Peleg et al., 2017). The fusion protein technology (fusion-tag technology) is one of the first approaches historically implemented to provide protein transport into the periplasmic space of *E. coli*. Although this approach was originally developed to facilitate protein detection and purification, it subsequently became clear that some of the fusion proteins were capable of imparting additional properties. For example, *Escherichia coli* maltose-binding protein (MBP) (Maina et al., 1988) and *Schistosoma japonicum* glutathione S-transferase (Smith and Johnson, 1988) as N-terminal fragments increase the chimeric protein yield (Butt et al., 1989), protect against intracellular proteolysis (Martinez et al., 1995; Jacquet et al., 1999), increase solubility (Pryor and Leiting, 1997; Kapust and Waugh, 1999) and even facilitate their folding (Waugh, 2005). The reasons why these proteins improve the solubility of their partners are not completely understood to date; however, the current knowledge is that polypeptide chains rich in positively charged amino acids help to overcome aggregation by increasing electrostatic repulsion among residues during translation. Additionally, the tag-fusion technology can prevent defective mRNA structures when fused to the N-terminal portion of the target sequence (Silva et al., 2021). However, all protein additives, whether large or small, can interfere to varying degrees with the protein structure and function (Goel et al., 2000; Bucher et al., 2002; Fonda et al., 2002; Chant et al., 2005). For this reason, it is usually recommended that fusion proteins are removed at the final step. In this case, the cleavage can be carried out at different stages. It can be done *in vitro*, after protein purification, or it can be cleaved *in vivo*, in cells. The latter option is preferable, primarily because it reduces the number of stages during protein purification (Silva et al., 2021). In addition to the highly specific proteases described and widely used, such as enterokinase, factor Xa (Kapust et al., 2002), SUMO protease (Butt et al., 2005) and thrombin (Esposito and Chatterjee, 2006) viral proteases such as tobacco etch virus (TEV) protease and human rhinovirus 3C protease play a special role in ensuring this stage.

The stringent specificity of viral proteases makes them an attractive tool for removing fusion proteins. The TEV protease is probably the most popular enzyme of this type. The cysteine protease TEV recognizes the amino acid sequence ENLYFQ↓G with high efficiency and cleaves the bond between Q (P1 position) and G (P1’ position) (Dougherty et al., 1989). Its stringent specificity, ease of production and high tolerance to amino acid residues at the P1’ position of its recognition site have contributed to its widespread use as an endoproteolytic reagent (Kapust et al., 2002; Tropea et al., 2009). Meanwhile, the inability to use the TEV protease for any reason (e.g., the presence of a proteolysis site within the protein sequence) requires an expansion of the protease arsenal to remove fusion proteins. The SARS-CoV-2 3CL main protease (Mpro) can be considered a candidate.

Like TEV protease, the coronavirus 3CL protease is a cysteine protease that “cuts” itself out of the polypeptide through self-activity, followed by formation of an active homodimer (Muramatsu et al., 2016). The active site of this enzyme includes conserved cysteine and histidine residues involved in acid-base catalysis (Xiong et al., 2021). The 3CL recognition site is highly conserved and includes glutamine and leucine/phenylalanine residues at positions P1 and P2, respectively, which correspond to the first and second residues upstream of the cleavage site on the polypeptide substrate, following the site is serine or alanine, L/FQ↓S/A, where ↓ marks the cleavage site (Ferreira and Rabeh, 2020).

The aim of this study was to test the feasibility of using the SARS-CoV-2 3CL protease in constructs enabling to cleavage of fusion proteins in the periplasmic space of *E. coli*. The results obtained may assist in the development of recombinant plasmid vectors containing the SARS-CoV-2 major protease gene for the production of soluble proteins in the *E. coli* system.

## 2 Materials and methods

### 2.1 Plasmid construction

Amplification of the GST nucleotide sequence was performed by PCR on the pGEX-T2 plasmid matrix using primers GST-3CL-F (5′-aaa​aaa​cat​atg​tcc​cct​cta​tag​gtt​tt-3′) and GST-3CL-R (5′-aaa​aaa​gga​tcc​ttg​ggt​gtc​cca​c-3′). The resulting nucleotide sequence was cloned into the pET21a vector using unique restriction sites (FauNDI/BamHI).

To amplify the nucleotide sequence of the coronavirus 3CL protease, OT-PCR was performed on the SARS-CoV-2 viral RNA matrix using BioMaster OT-PCR (Biolabmix LLC, Russia) and 3CL-F primers (5′-aaa​aaa​gga​tcc​agt​ggt​ttt​ttg​gaa​aaa​aat​ggc​att​cc-3′) and 3CL-R/3CL-6His-R (5′-aaa​aaa​gcg​gcc​ccg​ctt​aac​cgc​tac​cac​cgc​tta​ctg-3'/5′-aaa​aaa​gcg​gcc​ccg​ctt​agg​tgt​gtg​atg​tga​tga​ccc​cgc​tgc​ccc​ctg​ctg​aag​taa​cct​gac​ctg​act​g-3′). As a result, the nucleotide sequences 3CL and 3CL-6His were obtained and cloned at unique restriction sites (BamHI/CciNI) within the previously obtained pET21-GST vector.

In order to construct the pET21-GST-RBD plasmid, the RBD nucleotide sequence was amplified (308V-542N a.a.) using PCR on the pVEAL2-RBD plasmid matrix (employed previously in (Merkuleva et al., 2022) using primers csRBD_F (5′-aaa​aag​gat​gat​cca​cct​ctc​agc​tgc​tgt​ttt​ttt​ttt​ttt​ttg​taa​aaa​ggg​cat​cta​cca-3′) and IRES-RBD-R (5′-ggt​tgt​gtg​gtc​cat​att​cat​cgt​g-3′) and the GST nucleotide sequence using primers GST-3CL-F (5′-aaa​aaa​cat​atg​tcc​tcc​tat​act​agg​ttt​t-3′) and GST-3CL-R (5′-aaa​agg​tcc​ttg​tgg​tcg​cca​c-3′) on the pGEX-T2 matrix. The PCR product GST was treated with FauNDI and BamHI, and the PCR product RBD, with BamHI and SalI. Both products were then simultaneously cloned as part of the pET21a vector treated with FauNDI and SalI restrictases. The pET21-RBD plasmid obtained in this study was also used (Merkuleva et al., 2022).

The pNS-GST-3CL plasmid was produced by inserting the nucleotide sequence encoding GST-3CL fusion protein from pET21-GST-3CL into the pNS vector. The pNS vector is similar to the pET21 vector but contains the kanamycin resistance factor gene instead of the beta-lactamase gene. To construct the pET21a-GST-3CL-RBD plasmid, the nucleotide sequence of GST-3CL was amplified by PCR using primers Bse_3CL_R (5′-aaa​aac​ggg​ccg​act​gaa​ggt​aac​acc​gct​aca- 3′) and T7pro (5′-taa​tac​gac​tca​cta​tag​g-3′) on the pET21-GST-3CL plasmid matrix and the RBD fragment using primers Bse_RBD_F (5′-aaa​aac​ggg​ggt​gga​aag​ggc​atc​tac​ca-3′) and T7ter (5′-gct​agt​tat​tgc​tca​gcg​g-3′) on the pVEAL2-RBD matrix. The PCR product GST-3CL was treated with XbaI and BseX3I, and the PCR product RBD, with BseX3I and HindIII. Both products were then simultaneously cloned into the pET21a vector treated with XbaI and HindIII.

### 2.2 Producer preparation and protein expression

The target genes were expressed using *E. coli* strain BL21 (DE3). For this purpose, *E. coli* strain BL21 was chemically transformed with the developed vectors (pET21-GST-3CL, pET21-GST-RBD, pET21-GST-3CL-6His, pNS-GST-3CL, pET21-GST-3CL-RBD, and pET21-RBD). A self-made plasmid pET-TEV was used for TEV protease production.

Individual colonies carrying recombinant plasmids were cultured in lysogenic medium (LB) on an orbital shaker at 180 rpm and 37°C overnight. The inoculum was transferred into Erlenmeyer flasks containing fresh LB medium at a 1:100 ratio and cultured to an optical density of 0.8 (at 600 nm). Afterwards, isopropyl-β-D1-thiogalactopyranoside (IPTG) was added to a final concentration of 1 mM to induce cell culture, followed by culturing on an orbital shaker at 180 rpm, 37°C for 4 h. For culturing *E. coli* containing plasmids pET21-GST-3CL, pET21-GST-3CL-6His, pET21-GST-3CL-RBD, ampicillin (to a final concentration of 100 μg/mL), plasmids pNS-GST-3CL, kanamycin (to a final concentration of 50 μg/mL) were used as antibiotic, in case of two plasmids pNS-GST-3CL and pET21-GST-RBD, both antibiotics.

After culturing, the cell biomass was fractionated to separate the soluble proteins from the inclusion bodies. For this purpose, the cell biomass was precipitated by centrifugation at 5,000 g at 4°C for 20 min. The precipitate was resuspended in lysis buffer (20 mM Tris, 120 mM NaCl, 1 mM DTT, 0.1 mM EDTA) at a ratio of 20 mL buffer per 1 g biomass and incubated at 4°C for 30 min. At the end of incubation, cells were destroyed using a LUK-0.05/100-O ultrasonic homogenizer (Center for Ultrasound Technology, LLC, Russia) at 4°C. For this purpose, the cell suspension was treated three times with ultrasound (2000 W/L and 283 W/cm^2^) for 1 min and then cooled to 4°C. The inclusion cells were precipitated by centrifugation at 20,000 g for 20 min at 4°C and dissolved in PBS containing 8 M urea.

### 2.3 Purification of the target proteins

After culturing, the biomass from 1 L of culture medium was precipitated by centrifugation at 5,000×g for 20 min at 4°C. The bacterial precipitate was resuspended in 60 mL of lysis buffer (20 mM Tris-HCl, 0.12 M NaCl, 0.1 mM EDTA, 1 mM DTT, 15 mM imidazole, pH 7.5) and degraded on an ultrasonic disintegrator. The lysate was centrifuged at 16,000×g for 20 min at 4°C; the supernatant was applied to a Ni-Sepharose column filled with 2 mL of resin and pre-equilibrated with lysis buffer. The column was washed with 20 mL of wash buffer (20 mM Tris-HCl, 0.12 M NaCl, 20 mM imidazole, pH 7.5). The target protein was eluted with 3 mL of elution buffer (20 mM Tris-HCl, 0.12 M NaCl, 250 mM imidazole, pH 7.5). The eluate containing the target protein was dialyzed against 20 mL of buffer A (20 mM Tris-HCl, 0.12 M NaCl, pH 7.5).

### 2.4 SDS-PAGE and western blot

SDS-PAGE analysis of proteins was performed under denaturing conditions according to the Laemmli’s method using 10% and 15% gels. The gels were visualized by Coomassie staining. For western blot, proteins were transferred to a nitrocellulose membrane. The membrane was blocked with BSA, washed, and incubated in the presence of the 5000-fold diluted Anti-6X His tag antibody (ab1818184). Alkaline phosphatase-conjugated rabbit anti-mouse IgG antibodies were used as the secondary antibodies (Sigma, United States) with BCIP (5-bromo-4-chloro-3-indolyl phosphate) and NBT (nitro blue tetrazolium) as the substrates (ThermoFisher Scientific).

### 2.5 Analysis of 3CL enzyme activity

The enzyme activity of recombinant 3CL protease preparations was determined using a synthetic peptide substrate (Dabcyl-KTSAVLQSGFRKME-Edans). Fluorescence intensity was monitored using a CLARIOstar Plus instrument (BMG LABTECH, Germany) employing 355 nm and 460 nm wavelengths for excitation and emission, respectively. Reactive mixtures were prepared in a 384-well plate. All measurements were performed at 25°C. Each well contained one reaction mixture. The instrument was calibrated using the solution of the peptide that had undergone complete hydrolysis. The fluorescence value of this mixture was taken as 80%, according to the recommendations of a CLARIOstar Plus instrument. The reaction mixtures contained Tris-HCl buffer (pH = 7.3), 150 nM 3CL, and 100 µM substrate in the first well. The measurements were carried out in the kinetic scanning mode. The cycle time was 5 s and the number of cycles was 50. All experiments were repeated three times (n = 3). The companion MARS Data Analysis software (BMG LABTECH, Germany) was used for calculations.

For assessing the inhibitory capacity of compounds against the main coronavirus protease (3CL), the IC_50_ value was the half-maximal inhibitory concentration of a compound at which the fluorescence level is reduced by 50% compared to the value obtained without adding inhibitor. Reaction mixtures containing TrisHCl buffer (pH 7.3), EDTA, DTT, fluorogenic substrate, 3CL and test compound were prepared and incubated for 30 min in a 384-well plate at 30°C. The companion MARS Data Analysis software (BMG LABTECH, Germany) was used for IC_50_ calculations.

## 3 Results

### 3.1 Production of recombinant 3CL protease in soluble form

**FIGURE 1:**
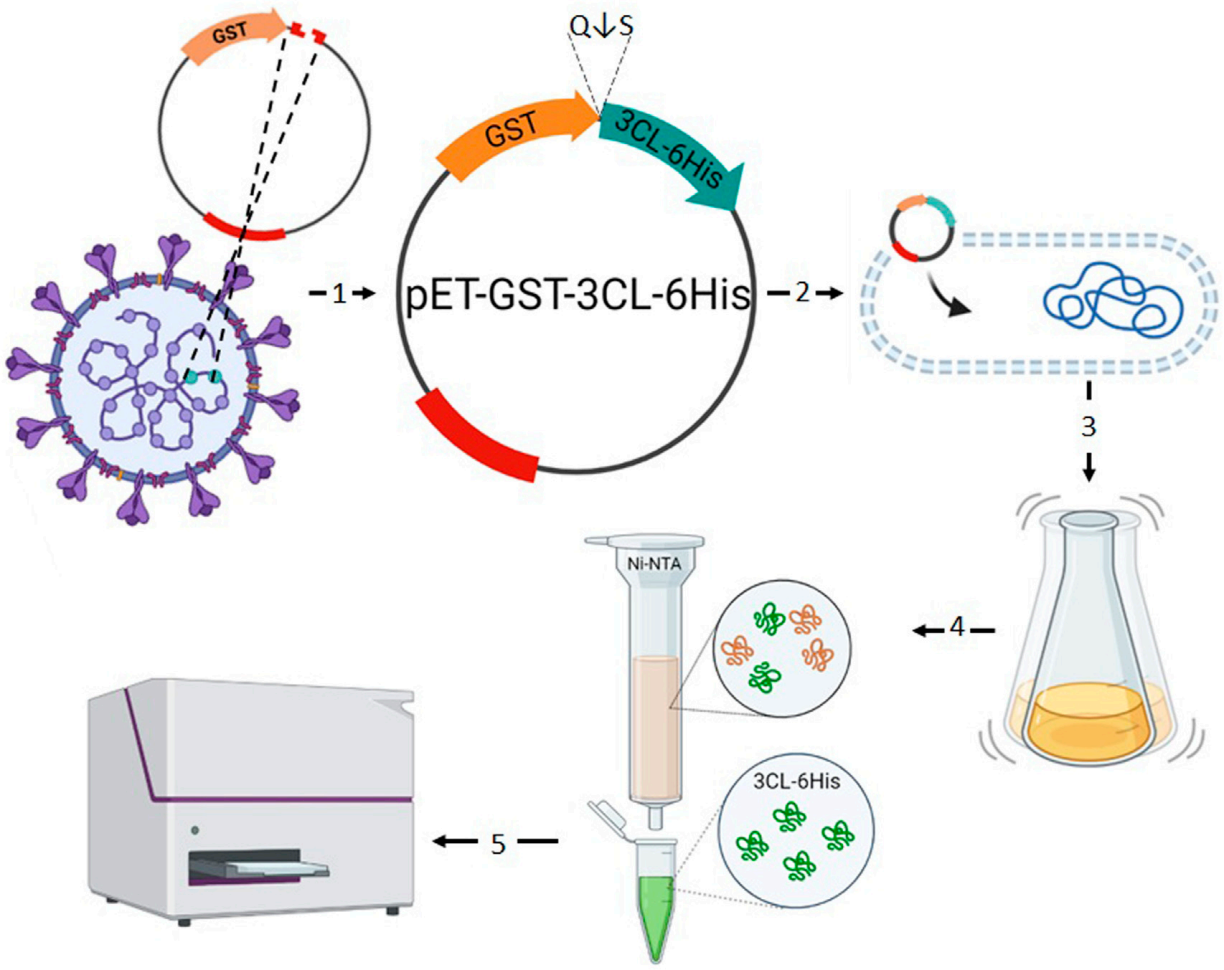
Outline of the first stage of the study. **1** - Recombinant plasmid pET21-GST-3CL-6His designed for synthesizing the SARS-CoV-2 main protease in a soluble form; **2** - Production of 3CL producer strain by transfection of *E. coli* cells. **3** - Cultivation of the producer strain; **4** - Chromatographic purification of the target protein. **5** - Verification of the functional activity of 3CL.

To obtain a preparation of recombinant 3CL protease, *E. coli* strain BL21 was chemically transformed with engineered plasmids pET21-GST-3CL or pET21-GST-3CL-6His (Figure 1 (1–2)) encoding 3CL fused with glutathione-S-transferase (GST). GST is a popular fusion protein for recombinant protein production as it increases protein solubility and promotes its transfer into the periplasmic space of *E. coli*, and can also be used as a tag for affinity chromatographic protein purification. In order to separate 3CL from GST, the amino acid sequence of GSTSAVLQ/SGFRK proteolysis site was included into the region separating them. After culturing the transformed colonies (Figure 1 (3–4)), the biomass of producer cells was fractionated to separate soluble proteins from the inclusion bodies, and the soluble fraction was purified chromatographically (Figure 1 (4–5)).

In order to assess the synthesis efficiency and identify the target protein location, the fractions obtained were analyzed electrophoretically in the presence of sodium dodecyl sulfate (SDS-PAGE) according to the Laemmli method (Laemmli, 1970) and by western blotting (Burnette, 1981).

**FIGURE 2:**
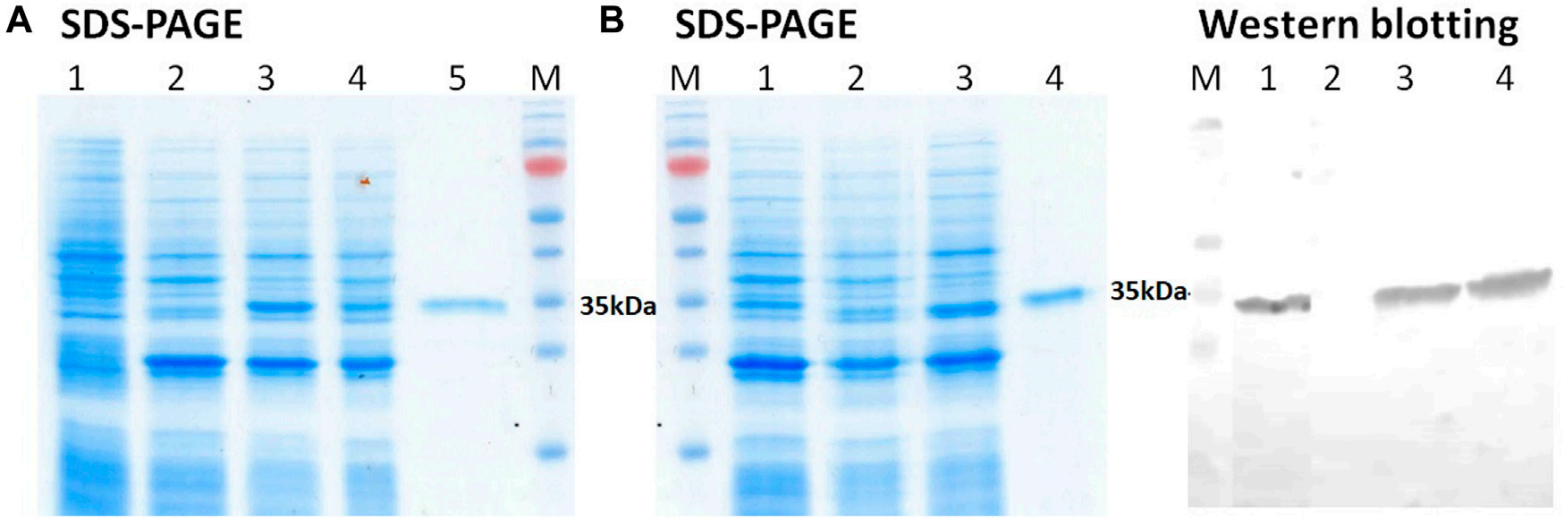
**(A)** SDS-PAGE analysis of *E. coli* BL21 (DE3) harboring pET21-GST-3CL plasmid: (1) non-transformed *E. coli* cells, *E. coli* - pET21-GST-3CL biomass (2), soluble (3), and insoluble (4) protein fractions, and purified 3CL (5); M - molecular weight markers. **(B)** SDS-PAGE and Western blot analyses of *E. coli* BL21 (DE3) harboring pET21-GST-3CL-6His plasmid: *E. coli* - pET21-GST-3CL-6His biomass (1), soluble (2), and insoluble (3) protein fractions, and purified 3CL-His (4); M - molecular weight markers. Proteins on the blot were probed with anti-His tag antibodies.

An analysis of the obtained biomass of *E. coli* cells containing plasmid pET21-GST-3CL-6His after IPTG induction (Figure 2A, lane 3, 6) showed the presence of two proteins whose electrophoretic mobility coincided with that calculated for 3CL (33.8 kDa) and GST (25.4 kDa). The content of the target protein was ≥5% of the total protein mass. Importantly, both proteins were detected in the soluble protein fraction (Figure 2A, lane 4, 8). To confirm this result, immunochemical identification was additionally performed by western blotting using anti-6His tag antibodies (Figure 2B). In turn, the inclusion body fraction does not contain 3CL (Figure 2A, lane 2, 7, Figure 2B, lane 7).

Thus, the developed vector pET21-GST-3CL-6His, when transfected with *E. coli*, provides synthesis of the target recombinant 3CL protease in a soluble form.

### 3.2 Authenticity analysis of the 3CL protease

**FIGURE 3:**
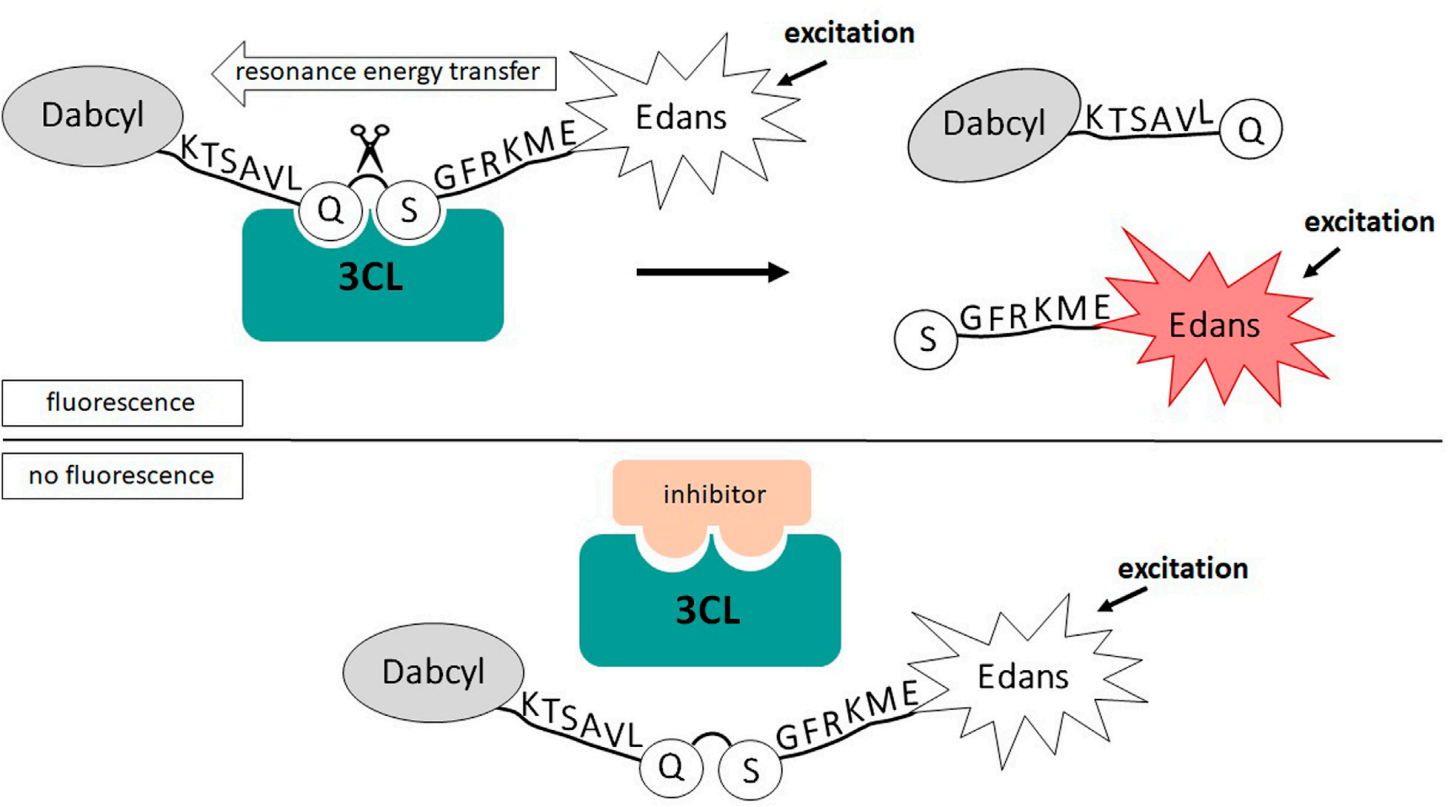
Schematic of 3CL activity analysis based on FRET resonant energy transfer.

In order to further verify the authenticity of the obtained protease, we decided to study its kinetic parameters and evaluate the effects of the known low-molecular-weight inhibitors. To determine the Michaelis constant (Km), the maximum reaction rate (Vmax), which were used to calculate the turnover rate of the enzyme (Kcat = Vmax/[E]) and its catalytic efficiency (Kcat/Km) we used a popular approach based on the use of fluorogenic peptide substrate simulating 3CL proteolysis site (Figure 3).

A recombinant TEV protease obtained by us, as well as a commercially available one (TEVp, Biolabmix, Russia), were used as a reference (Table 1). This protease was chosen in our work because it shows exquisitely high specificity, is relatively easy to make in large quantities. It is the most common control in studies and its use allows for broad comparisons (Esposito and Chatterjee, 2006). The peptide substrates for 3CL and TEV proteases had the same fluorophore/quencher pair and differed only in the consensus region of the proteolysis site. This synthetic peptide design allowed us to measure the kinetic parameters for 3CL and TEV proteases under identical conditions.

**TABLE 1:** The kinetic parameters of recombinant proteases.

Рrotease	Km, µM	Kcat, s^-1^	Catalytic efficiency (kcat/Km), µM^−1^ s^−1^
3CL	17.67 ± 1.93	2.99 ± 0.49	0.17 ± 0.02
TEV	23.49 ± 4.68	0.30 ± 0.03	0.013 ± 0.003
TEVр (Biolabmix)	22.08 ± 1.12	0.40 ± 0.09	0.018 ± 0.004

Km, kcat and kcat/Km data are shown as mean, ± standard deviation (*n* = 3).

The following values of kinetic parameters were obtained: Km = 17.67 ± 1.93 µM; Kcat = 2.99 ± 0.49 s^-1^, and Kcat/Km = 0.17 ± 0.02 µM^-1^s^-1^. The resulting Michaelis constant agreed with the data reported earlier (Tripathi et al., 2020; Jin et al., 2020).

The values of the kinetic parameters obtained for the TEV protease are consistent for the commercial and laboratory samples, as well as agree with the published values (Kraft et al., 2007). Meanwhile, the results show that the catalytic efficiency of 3CL is one order of magnitude higher than that of the widely used TEV protease.

To further evaluate the authenticity of the enzyme that we obtained using the prokaryotic expression system, we analyze a sensitivity of 3СL to inhibitors described in the literature (disulfiram, ebselen, GC376 and ML188). To do this, we used the method developed by us earlier based on measuring fluorescence without adding the inhibitor and in the presence of the inhibitor (Figure 3; Shcherbakov et al., 2022).

**FIGURE 4:**
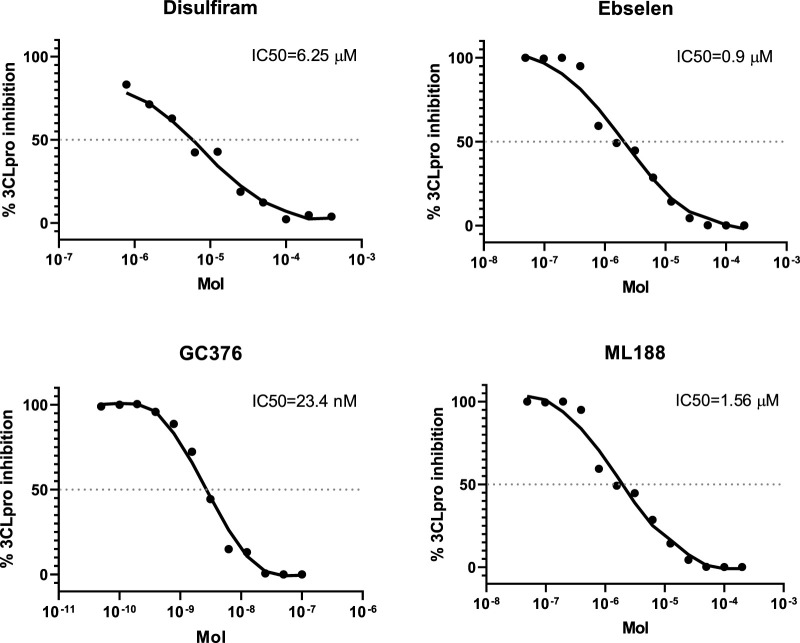
The concentration-response curves for the known 3CL protease inhibitors.

The IC_50_ values obtained in the study for disulfiram, ebselen, GC376 and ML188 were 6.25 ± 1.97, 0.9 ± 0.4 µM, 23.4 ± 4.5 nM and 1.56 ± 0.3 nM respectively. The results obtained are consistent with the results published earlier (Jin et al., 2020), (Lockbaum et al., 2021; Zakharova et al., 2021) (Figure 4), which confirms that 3СL retains its properties when expressed in *E. coli* cells. The use of this test system makes it possible both to detect new inhibitors of the main viral protease (Baev et al., 2022) and to study the mechanism of action of compounds that have shown activity against the infectious SARS-CoV-2 virus (Filimonov et al., 2022).

The combination of the results indicates that the protease obtained in our study was indeed a SARS-CoV-2 protease. This enzyme was similar to the most popular protease TEV of researchers in its characteristics, but has an order of magnitude higher catalytic activity.

### 3.3 The use of the 3CL protease SARS-CoV-2 for cleavage fusion proteins in the periplasmic space of *E. coli*

The catalytic mechanism of polyprotein cleavage by 3CL includes autoprocessing and autolytic cleavage steps. We hypothesized that this property of 3CL allows one to use it not only for *in vitro* cleavage in tube but as a tool for *in vivo* cleavage of fusion proteins in *E. coli* cells. In order to test this hypothesis, the receptor-binding domain (RBD) of the S-protein SARS-CoV-2, which was previously obtained only in an insoluble form from *E. coli* inclusion bodies by us and other research groups, was chosen as a model protein (Fitzgerald et al., 2021; He et al., 2021; Merkuleva et al., 2022).

**FIGURE 5:**
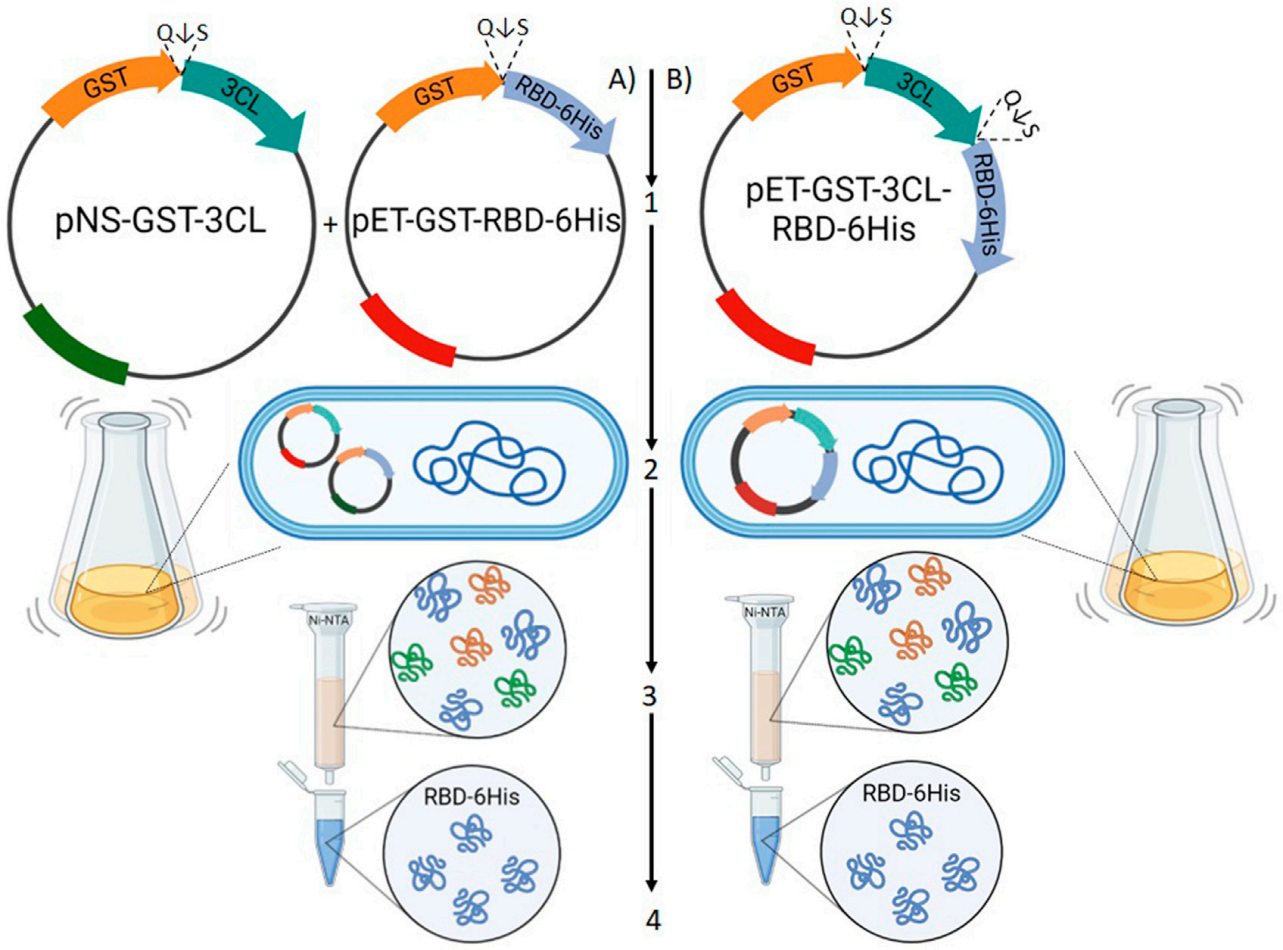
A schematic diagram of testing the use of 3CL as a tool to remove fusion proteins *in vivo* in *E. coli* cells. Option **(A)** the scheme of simultaneous transformation of two plasmids, one encoding 3CL, the other one encoding RBD fused to GST with a 3CLpro proteolysis site (Q↓S) between them. Option **(B)** Transfection scheme with one plasmid encoding GST, 3CL and RBD. (**1**) Construction of recombinant plasmids that provide RBD synthesis in a soluble form. (**2**) Cultivation of recombinant strains. (**3**) Purification of the target protein. (**4**) Verification of functional activity of RBD.

Our idea was to use a system consisting of two plasmids: one encoding 3CL and the other one encoding RBD fused to GST with a 3CL proteolysis site (SAVLQ↓SGF) between them (Figure 5A).

We constructed a pET21-GST-RBD plasmid encoding SARS-CoV-2 (Wuhan-Hu-1) RBD fused with GST at the N-terminus and 6X His tag at the C-terminus. The pNS-GST-3CL plasmid, which encodes a GST-3CL fusion protein and, unlike pET21-GST-RBD, carries resistance to the antibiotic kanamycin to ensure selection of double transformants (Figure 5A (1)), was also obtained.

**FIGURE 6:**
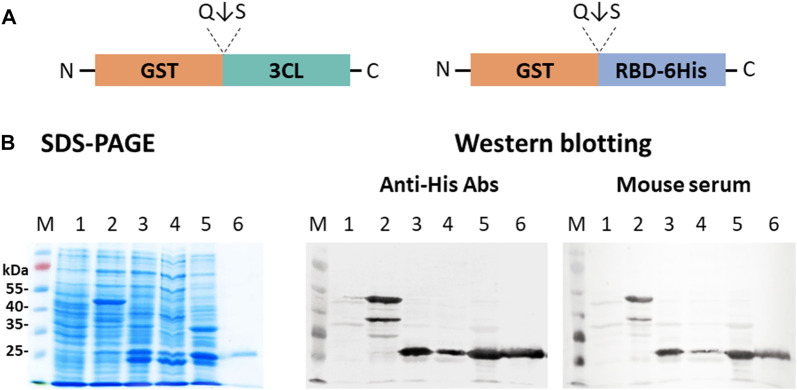
Expression of pET21-GST-RBD and pNS-GST-3CL plasmids in *E. coli* BL21 (DE3) cells: **(A)** Schematic representation of the structure of proteins encoded by pET21-GST-RBD and pNS-GST-3CL. **(B)** SDS-PAGE and western blot analyses of *E. coli* BL21 harboring pNS-GST-3CL or both pNS-GST-3CL and pET21-GST-RBD plasmids: M - molecular weight marker; (1) non-transformed *E. coli* biomass; (2) *E. coli* cells + pNS-GST-3CL; (3) *E. coli* + pET21-GST-RBD + pNS-GST-3CL, (4) soluble and (5) insoluble protein fractions; (6) purified RBD. Proteins on the blot were probed with anti-His tag antibodies or mouse hyperimmune serum.

*E. coli* BL21 (DE3) cells were transfected with plasmid pET21-GST-RBD or simultaneously with plasmids pET21-GST-RBD and pNS-GST-3CL. Biomass generation, induction with 1M IPTG, and separation of cell fractions were performed according to the procedure described above. Proteins of the obtained fractions were analyzed by 10% SDS-PAGE and immunoblotting using anti-His tag antibodies and serum from a hyperimmune mouse immunized with RBD obtained in mammalian cells CHO-K1 (Merkuleva et al., 2022) (Figure 6).

A major product (∼50 kDa) corresponding to the fusion protein GST-RBD (theoretical weight of 54.4 kDa) was observed in cells transformed only with pET21-GST-RBD. And also lower molecular weights by-products were observed, which were also specifically stained with anti-His antibodies to the tag and RBD-specific mouse hyperimmune serum (Figure 6, lane 2). The products corresponding to RBD, GST and 3CL (∼26 kDa, ∼25 kDa, ∼34 kDa) were detected in cells upon co-expression of pET21-GST-RBD and pNS-GST-3CL. Moreover, the target protein RBD was present in the soluble protein fraction (Figure 6, lane 4).

**FIGURE 7:**
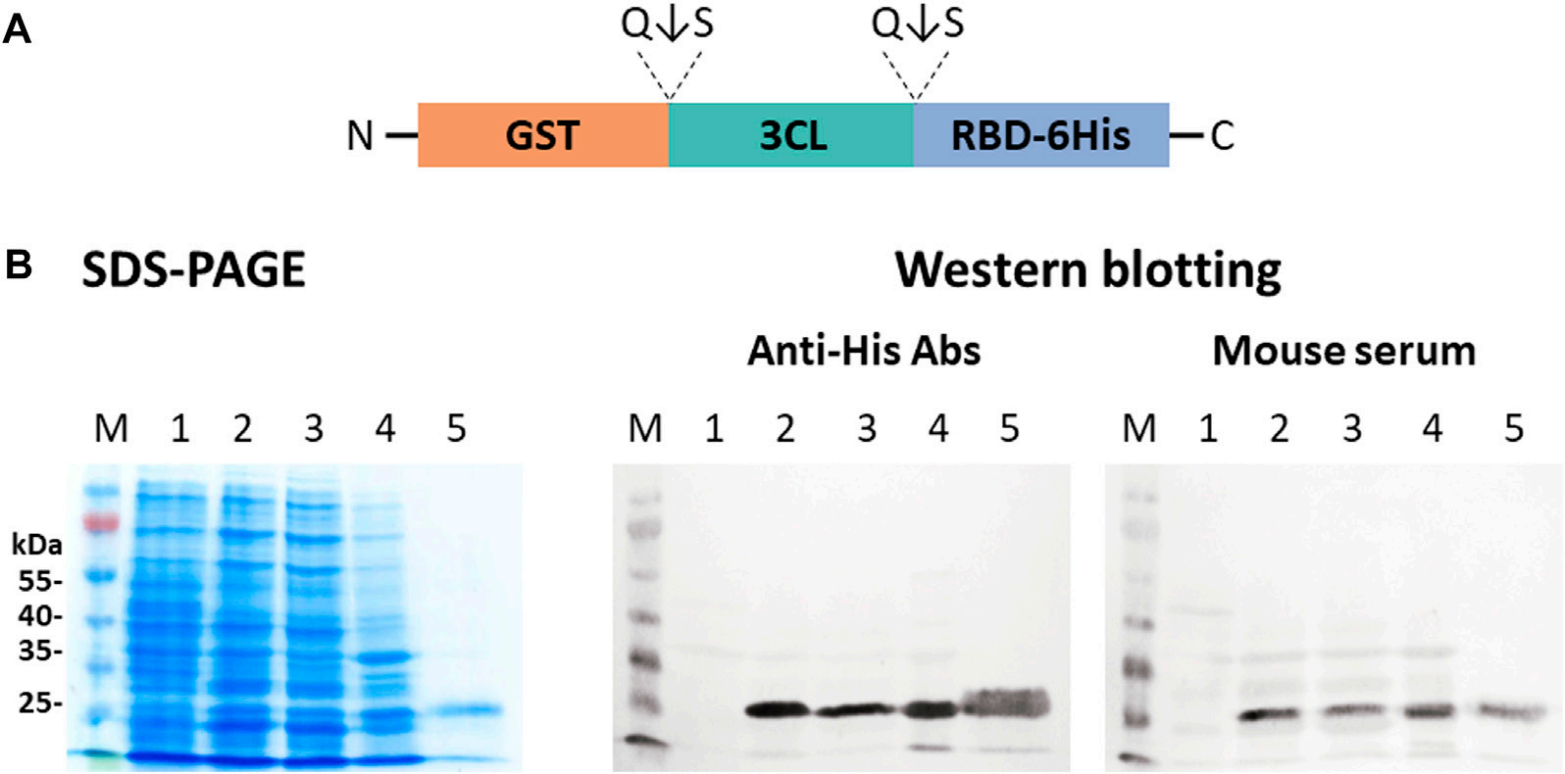
Expression of the pET21-GST-3CL-RBD plasmid in *E. coli* BL21 (DE3) cells: **(A)** A sche-matic diagram of the protein structure encoded by pET21-GST-3CL-RBD. **(B)** SDS-PAGE and western blot analyses of *E. coli* BL21 harboring pET21-GST-3CL-RBD plasmid: M—molecular weight marker; (1) non-transformed *E. coli* biomass; (2) *E. coli* + pET21-GST-3CL-RBD, (3) soluble and (4) insoluble protein fractions; (5) purified RBD. Proteins on the blot were probed with an-ti-His tag antibodies or mouse hyperimmune serum.

The use of a single vector instead of a two-vector system may be a more attractive strategy (Figure 5B). It increases the efficiency of obtaining transformants and does not require double selection using two antibiotics. We, therefore, obtained the plasmid pET21-GST-3CL-RBD in which 3CL was located between the GST fusion protein and the target RBD. This concept involves expression of the GST-3CL-RBD polypeptide from which the 3CL protease is autocatalytically excised/leached releasing the target RBD protein. When the pET21-GST-3CL-RBD plasmid was expressed in *E. coli* BL21 (DE3), we observed proteins corresponding to GST and 3CL, as well as accumulation of the target protein RBD in soluble form (Figure 7).

Therefore, both proposed concepts of fusion protein removal with 3CL protease in *E. coli* cells showed their effectiveness in producing the target protein in a soluble form into the periplasmic space of *E. coli* cells.

## 4 Discussion

Finding ways to produce a target recombinant protein of interest in a soluble form in the *E. coli* expression system is perhaps the challenge faced by researchers dealing with genetic engineering and the need to produce various proteins, bacterial, viral, plant, etc. Researchers already have many tools at their disposal, but it is not uncommon to find new ones or adapt the old tools when facing new and non-standard challenges. The last striking example of such a protein that is so difficult to obtain in *E. coli* is RBD of spike protein SARS-CoV-2. In our previous work, we failed to obtain soluble RBD in the *E. coli* expression system. Other researchers have encountered similar difficulties (Fitzgerald et al., 2021; He et al., 2021; Merkuleva et al., 2022).

In this work, we decided to use an *in vivo* variant of the fusion protein technology to obtain soluble RBD. Despite the fact that this technology is not new, its improvement is an important task, since it allows expanding the arsenal of tools for obtaining recombinant proteins. In particular, it is important to expand the list of proteases that can be used for cleavage. At the same time, it is necessary that the protease retain its activity when expressed in *E. coli*, and have proteolytic stringency, so it must rarely cut in nonspecific sites within the target protein (Silva et al., 2021). As a protein providing delivery of the fusion protein to the *E. coli* periplasm, we chose GST. This protein is often successfully used for expression different proteins (Esposito and Chatterjee, 2006).

We decided to use 3СL as the cleavage enzyme. The use of 3СL, according to our data, is the first case of using this SARS-CoV-2 enzyme for these purposes. Moreover, this is the first time that coronavirus proteases have been used for this purpose. In the beginning, we focused on the study of 3СL obtained in *E.сoli*. Previously, 3СL was repeatedly obtained in this expression system, mainly for the search for inhibitors (Jin et al., 2020). However, in our work, we had a different goal, to use the gene of this enzyme as a component of protein fusion technology. As a control, we used the TEV protease, one of the most popular proteases for researchers (Kapust and Waugh, 2000; Sanchez and Ting, 2020). According to our data, for the first time we have carried out their direct comparison using a unified approach. Peptide substrates of similar design and carrying the same fluorophores were used. It is interesting that we found that 3СL has values of K_m_ close to TEV, but has an order of magnitude higher catalytic efficiency (Table 1). These results are consistent with the published results, in particular, it was noted that 3СL SARS-СoV-2 has a higher catalytic efficiency than 3СL SARS-СoV (Kraft et al., 2007). The use of known 3CL inhibitors allowed us to confirm the identity of the obtained enzyme. The limitation of this part of our work is that we have not studied in detail the specificity of 3CL SARS-CoV-2 proteolysis; we plan to carry out this work in the following works.

Further, on the basis of the GST, RBD, and 3CL genes, we designed plasmids capable of providing the synthesis and transport of chimeric proteins into the periplasmic space of *E. coli* (Figure 5)**.** Initially, a two-plasmid scheme was used. In this case, the synthesis of the GST-RBD and GST-3СL products was carried out separately; autocatalytic cleavage of 3CL followed by GST-RBD cleavage occurred in the periplasm, which we recorded using SDS-PAAG and immunoblotting (Figure 6). However, the use of two plasmids has difficulties associated with both the need to use two antibiotics and the low efficiency of transformation. Therefore, it was decided to construct a plasmid encoding GST-3CL-RBD fusion protein (Figure 5). Transformation of the cells with the resulting plasmid indeed made it possible to obtain soluble RBD in *E. coli*. (Figure 7). It should be noted that both in the case of the scheme with two plasmids and with one, the efficiency of obtaining soluble RBD was not 100%, part of the protein was found in an insoluble form. Further work will be aimed to optimizing the cultivation conditions, which we believe can increase soluble protein production efficiency. It is also a limitation of our work is that we did not use other variants of fusion proteins, such as MBP or Trx (Esposito and Chatterjee, 2006), which would allow us to generalize our approach. Such studies can be carried out in the future. But in this study, it was important for us to demonstrate the innovative concept of using the 3CL coronavirus protease.

## 5 Conclusion

In this study, we demonstrated the possibility of using recombinant 3CL protease as an agent for cleaving fusion proteins. Using resonant energy transfer FRET, it was shown that the recombinant 3CL produced in *E. coli* retains its specific biochemical properties. Moreover, a comparative analysis using a similar substrate showed that the enzyme we obtained has a catalytic efficiency more than one order of magnitude higher than the commonly used TEV protease. The work illustrates that the incorporation of the 3CL gene into recombinant plasmids made it possible to construct *E. coli* strains in which fusion proteins containing RBD were synthesized and transported into the periplasmic space of the cells. In the periplasmic space, RBD was quantitatively released from the chimeric protein by the 3CL protease. The resulting RBD protein not only retained its solubility, but was also recognized by specific monoclonal and polyclonal antibodies. Our future studies will be aimed at confirming the correctness of hydrolysis of fusion proteins by 3CL protease using chromatography–mass spectrometry methods. In addition, we will compare the developed platform with other existing analogues. The results obtained in the work allow expanding the repertoire of specific proteases for researchers and biotechnologists.

## Data Availability

The original contributions presented in the study are included in the article/Supplementary Material, further inquiries can be directed to the corresponding authors.
