# Corrigendum: The main protease 3CLpro of the SARS-CoV-2 virus: how to turn an enemy into a helper

**DOI:** 10.3389/fbioe.2023.1294266

**Published:** 2023-11-06

**Authors:** Svetlana V. Belenkaya, Iuliia A. Merkuleva, Olga I. Yarovaya, Varvara Yu Chirkova, Elena A. Sharlaeva, Daniil V. Shanshin, Ekaterina A. Volosnikova, Sergey Z. Vatsadze, Mikhail V. Khvostov, Nariman F. Salakhutdinov, Dmitriy Shcherbakov

**Affiliations:** ^1^ Laboratory of Bionanotechnology, Microbiology and Virology, Novosibirsk State University, Novosibirsk, Russia; ^2^ State Research Center of Virology and Biotechnology VECTOR, Koltsovo, Russia; ^3^ Department of Medicinal Chemistry, N.N Vorozhtsov Novosibirsk Institute of Organic Chemistry SB RAS, Novosibirsk, Russia; ^4^ Department of Physical-Chemistry Biology and Biotechnology, Altay State University, Barnaul, Russia; ^5^ N.D Zelinsky Institute of Organic Chemistry, Russian Academy of Sciences, Moscow, Russia

**Keywords:** *E. coli* bacteria, 3CL protease, TEV protease, RBD, SARS-CoV-2

In the published article, there was an error in the **Materials and methods** section at the *Paragraph 2.2*. Plasmid name was displayed as “A commercially available plasmid pRK793 was used for TEV protease production”.

The correct statement is “A self-made plasmid pET-TEV was used for TEV protease production”.

The authors apologize for this error and state that this does not change the scientific conclusions of the article in any way. The original article has been updated.

